# Supplementary material for: Multilayered control of splicing regulatory networks by DAP3 leads to widespread alternative splicing changes in cancer
Source: Nat Commun. 2022 Apr 4;13:1793. doi: 10.1038/s41467-022-29400-7 (PMC8980049; doi:10.1038/s41467-022-29400-7)
Supplement: Supplementary file 1 — Supplementary Information [file 41467_2022_29400_MOESM1_ESM.pdf]

Multilayered Control of Splicing Regulatory Networks by DAP3 Leads to Widespread Alternative Splicing Changes in Cancer

Supplementary Information: Supplementary discussion and supplementary figure 1-11.

Supplementary Discussion

Confirmation of the specificity of the DAP3 eCLIP-Seq analysis

To ensure the specificity of the DAP3 eCLIP-Seq analysis, we examined the presence of two well-known spliceosomal RBPs U2AF35 and U2AF65 as well as two validated DAP3 interactors SFPQ and NONO (Fig. 3a) in the DAP3 immunoprecipitates and found no presence of these four proteins in the IP eluates after harsh washing in the eCLIP experiments (Supplementary Fig. 1a and b). These data suggested that when eCLIP-Seq was carried out, the wash condition was harsh enough to remove DAP3 interactors and other RBPs which may potentially bind to the target RNAs.

Confirmation of an enrichment of DAP3 binding to its splicing targets by a randomization analysis

To confirm there is a significant enrichment of DAP3 binding peaks in genes undergoing DAP3-modulated splicing ("DAP3-modulated AS genes"; n=3,262) when compared to genes without DAP3-modulated splicing ("Non-modulated AS genes"; n=9,828), we accounted for two factors "expression levels" and "sample size" in the comparison by conducting a further randomization analysis. First, we checked the expression levels of these two groups of genes and found there was no significant difference in expression levels of DAP3-modulated and non-modulated AS genes (Supplementary Fig. 4c). Next, we randomly selected 3,262 genes out of 9,828 non-modulated AS genes, followed by the analysis of the number of bound genes (the analysis was repeated 100 times) (Supplementary Fig. 4d). When compared to 2,180 bound genes in the "DAP3-modulated AS genes" group, an average of ~1,700 bound genes found in the "non-modulated AS genes" group confirmed the enrichment of DAP3 binding to its splicing targets.

a

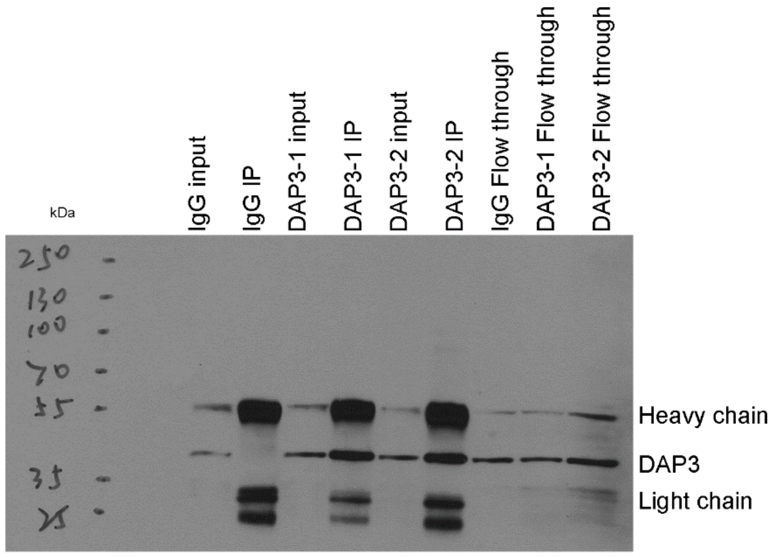

b

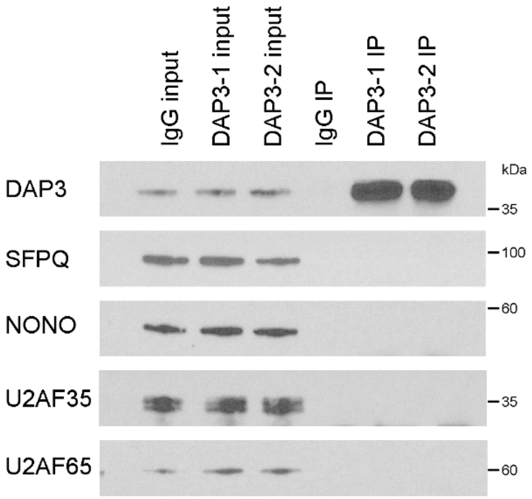

Supplementary Figure 1

Supplementary Figure 1. Western blot analysis of DAP3 eCLIP samples.

(a) EC109 cells were UV crosslinked, fragmented and immunoprecipitated using control IgG or a specific antibody against DAP3. The "input", immunoprecipitated (IP) and flow through samples were subjected to gel electrophoresis and membrane transfer for western blot analysis.

(b) Western blot analyses of splicing factors SFPQ, NONO, U2AF35 and U2AF65 in the DAP3 eCLIP samples. DAP3-1 and DAP3-2 are two eCLIP biological replicates.

Source data are provided in Source Data file.

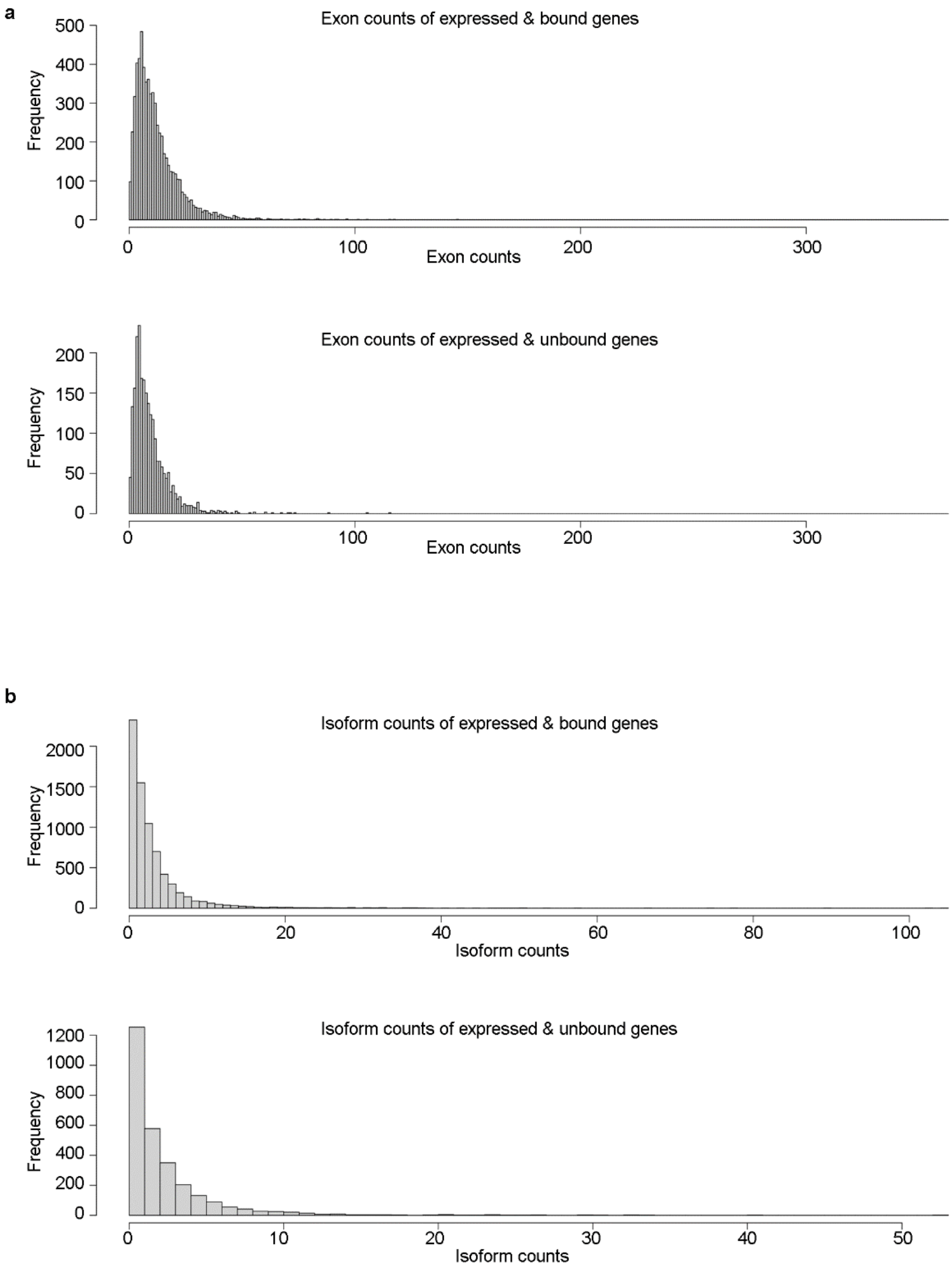

Supplementary Figure 2

Supplementary Figure 2. Comparative analysis of gene structure between genes expressed and bound by DAP3 and those expressed but not bound by DAP3.

Bar charts showing the frequency of DAP3-bound or unbound expressed genes (*y*-axis) with the indicated number of exons (a) or isoforms (b) per gene (exon counts or isoform counts; *x*-axis) in EC109 cells.

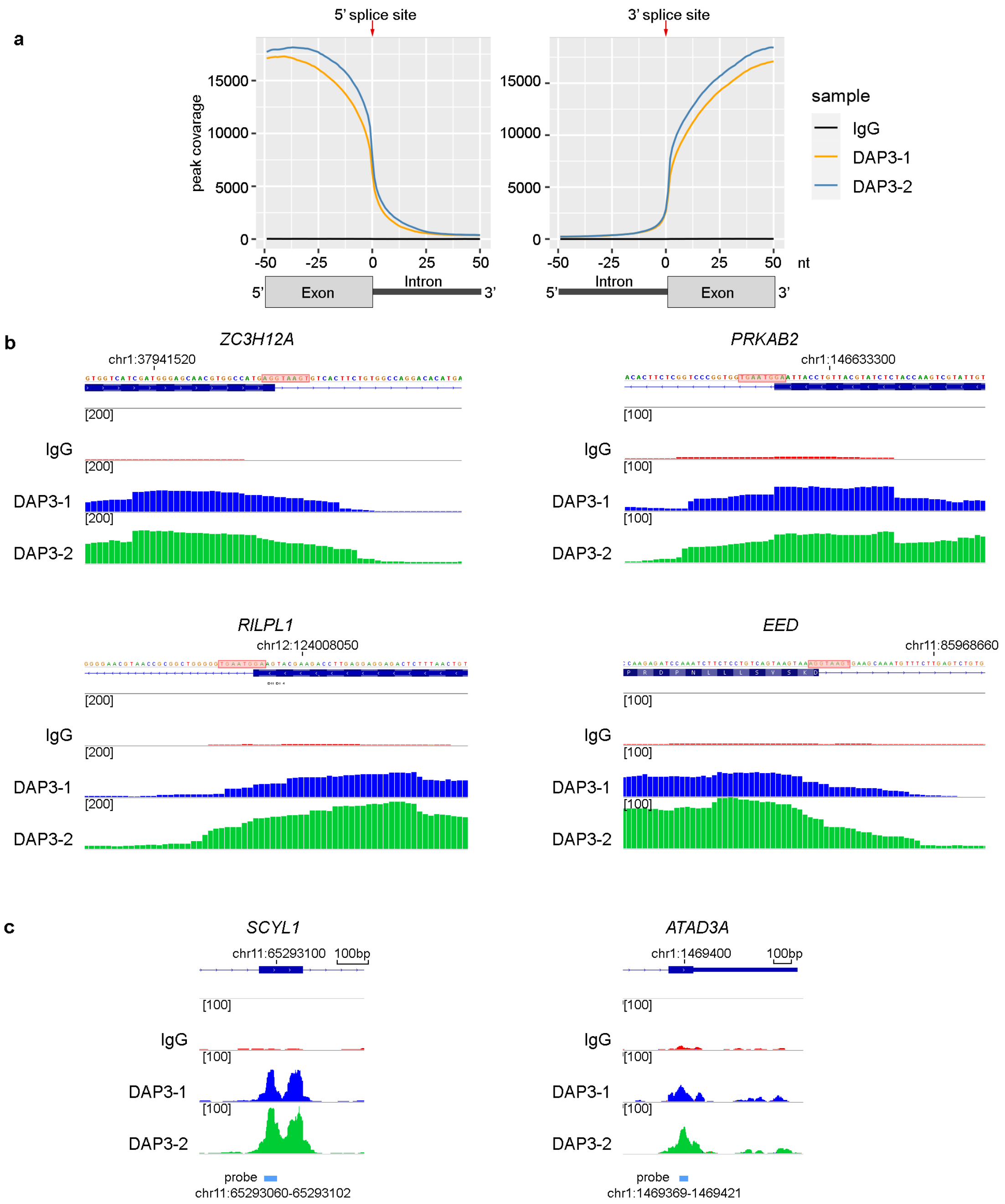

Supplementary Figure 3

Supplementary Figure 3. Integrated genome viewer (IGV) browser tracks of the DAP3 eCLIP peaks with top ranked binding motifs in representative target genes.

(a) DAP3 eCLIP peak coverage at -50 to 50 nucleotides (nt) around 5' and 3' splice sites.

(b) IGV browser tracks of the DAP3 eCLIP peaks spanning the 5' splice site junction with AGGUAAGU motifs on *ZC3H12A*, *PRKAB2*, *RILPL1* and *EED* gene. DAP3-binding motif AGGUAAGU is indicated by box.

(c) IGV browser tracks of the DAP3 eCLIP peaks spanning the genomic loci of *SCYL1* and *ATAD3A* gene. The positions of RNA probe sequences used in REMSA are indicated.

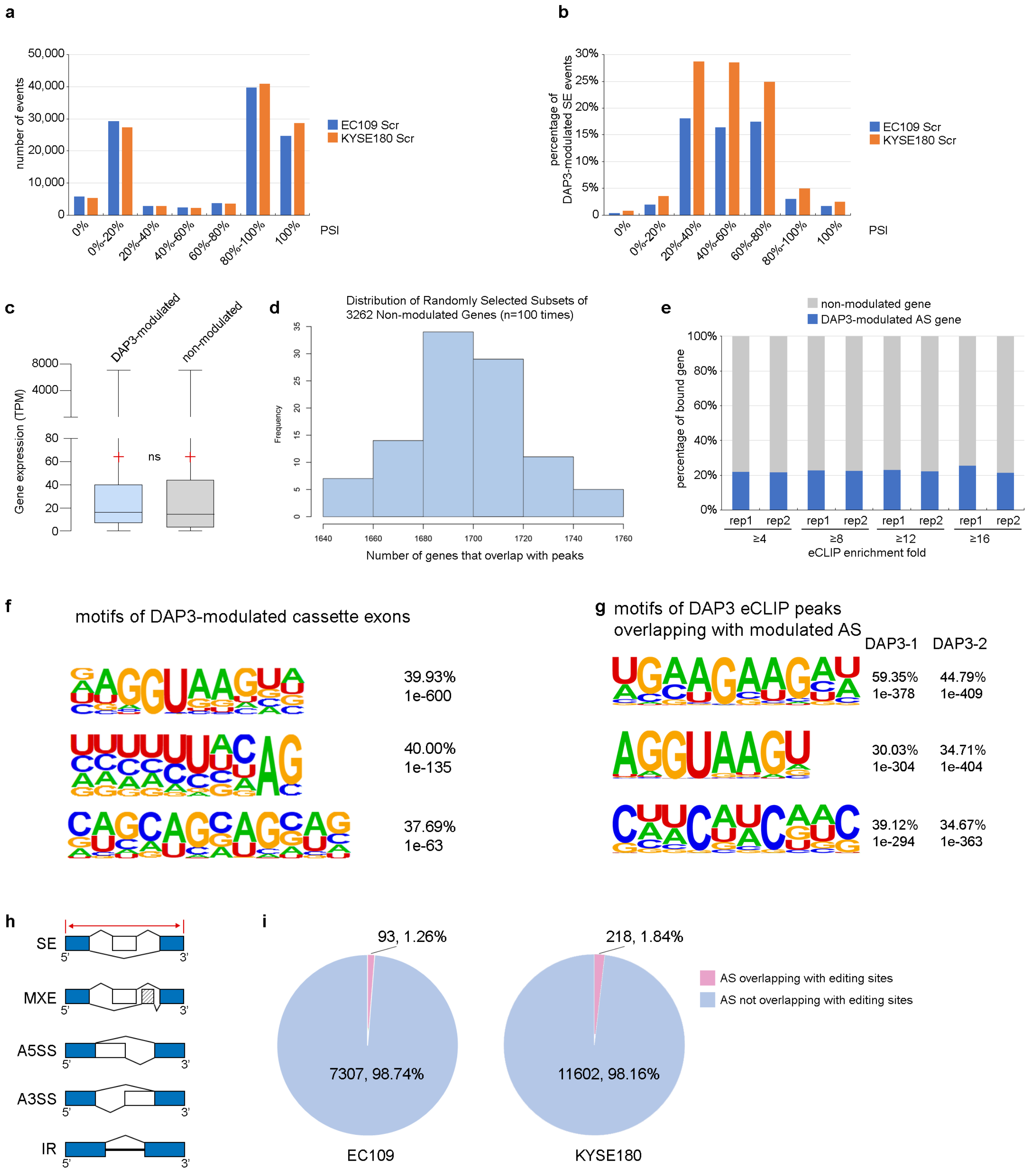

Supplementary Figure 4

Supplementary Figure 4. Analysis of the DAP3 binding peaks and DAP3-modulated splicing events, from DAP3 eCLIP-Seq and RNA-Seq datasets respectively.

(a) Bar chart showing the number of SE events in the indicated groups based on the basal PSI values in EC109 and KYSE180 scramble control cells.

(b) Bar chart showing the percentage of DAP3-modulated SE events in the indicated groups.

(c) Expression levels (TPM) of genes in the "DAP3-modulated genes" (n= 3,262) and "Non-modulated genes" (n= 9,828) groups. Data are presented as box plots with median (horizontal line), mean ('+'), 25–75 percentile (box), and min to max values (whisker) for each group (Unpaired Welch's *t*-test, two-sided; ns, not significant).

(d) Bar chart showing the frequency distribution of gene sets (n=100) with the indicated number of DAP3 eCLIP peaks. Each gene set comprised randomly selected 3,262 genes from the 9,828 non-modulated genes.

(e) Percentages of DAP3-bound genes that underwent or did not undergo DAP3-modulated splicing ('DAP3-modulated AS gene' and 'non-modulated gene', respectively) in the indicated groups based on the fold enrichment of eCLIP peaks. rep1, rep2: eCLIP biological replicate 1 and 2.

(f) Top 3 enriched motifs in sequences of DAP3-modulated cassette exons.

(g) Top 3 enriched motifs in DAP3 eCLIP peak sequences within the region from the upstream to downstream constitutive exon of DAP3-modulated splicing event (as indicated in (**h**)).

(h) Schematic diagram illustrating 5 types of alternative splicing events. Editing sites residing in the indicated region (red arrow; its boundaries are indicated red vertical lines) were included for the analysis described in (**i**).

(i) Pie charts showing the proportion of DAP3-modulated splicing events with or without DAP3-regulated A-to-I editing sites within the indicated region.

Source data are provided in Source Data file.

a

DAP3-modulated splicing in EC109

Go term	p-value
DNA replication(GO:0006260)	3.84e-09
spindle organization(GO:0007051)	1.59e-07
mitotic cell cycle phase transition(GO:0044772)	5.74e-07
microtubule organizing center organization(GO:0031023)	1.10e-06
chromosome segregation(GO:0007059)	3.37e-06
base-excision repair(GO:0006284)	4.00e-06
cilium organization(GO:0044782)	4.32e-06
regulation of mRNA metabolic process(GO:1903311)	6.93e-06
mRNA processing(GO:0006397)	9.20e-06
microtubule cytoskeleton organization involved in mitosis(GO:1902850)	1.27e-05

b

DAP3-modulated splicing in KYSE180

Go term	p-value
DNA replication(GO:0006260)	4.94e-13
mRNA processing(GO:0006397)	1.16e-12
mitotic cell cycle phase transition(GO:0044772)	1.16e-12
RNA splicing(GO:0008380)	4.63e-12
regulation of cell cycle phase transition(GO:1901987)	1.35e-10
covalent chromatin modification(GO:0016569)	1.74e-10
double-strand break repair(GO:0006302)	6.37e-10
proteasomal protein catabolic process(GO:0010498)	1.00e-08
organelle fission(GO:0048285)	3.03e-08
cilium organization(GO:0044782)	4.35e-08

Supplementary Figure 5

Supplementary Figure 5. GO analysis of genes undergoing DAP3-modulated splicing.

The top 10 most enriched biological processes of genes undergoing DAP3-modulated splicing in (a) EC109 and (b) KYSE180 cells are shown.

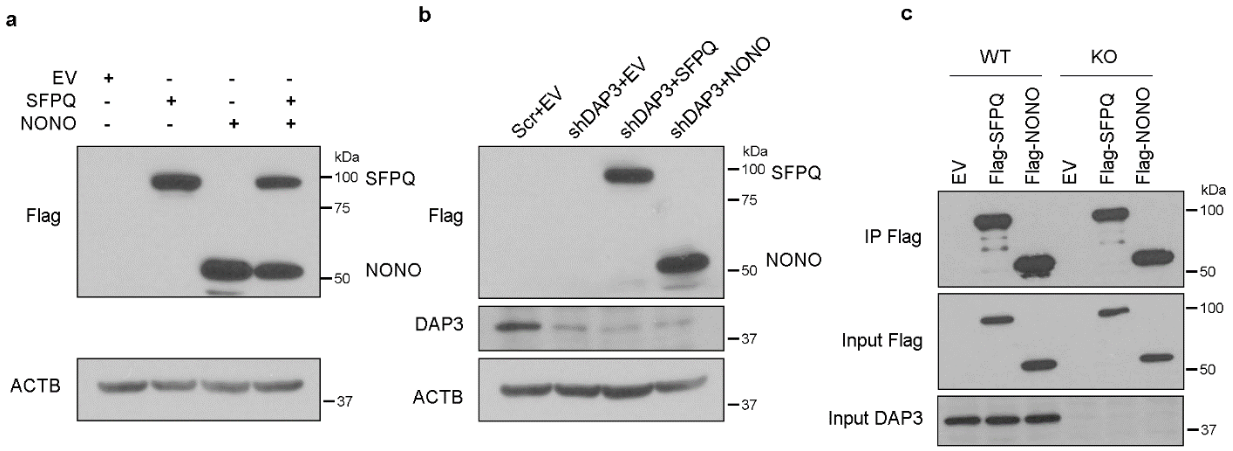

Supplementary Figure 6

Supplementary Figure 6. DAP3 facilitates the interaction of splicing factors SFPQ and NONO to target RNAs.

(a) Western blot analysis of Flag-tagged SFPQ and NONO overexpression in EC109 cells.

(b) Western blot analysis of Flag-tagged SFPQ and NONO overexpression in DAP3 depleted EC109 cells.

(c) Western blot analysis of SFPQ and NONO RIP-qPCR samples**.** The input and IP samples of Flag-SFPQ and NONO RIP experiments were analyzed by western blot to examine pull down efficiency.

Source data are provided in Source Data file.

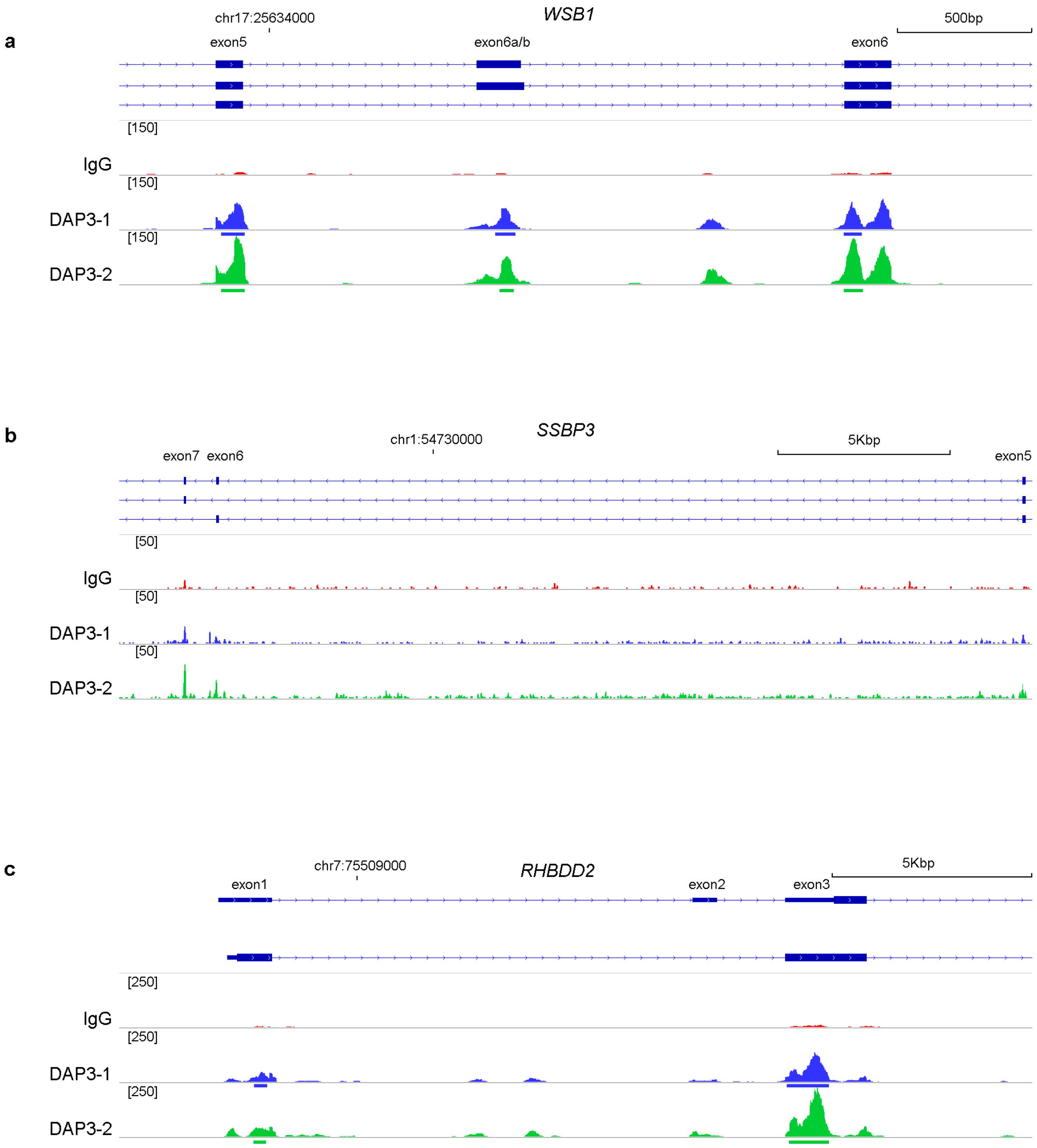

Supplementary Figure 7

Supplementary Figure 7. DAP3 binding peaks on *WSB1*, *SSBP3* and *RHBDD2* gene, from the eCLIP-Seq data.

(a) IGV browser tracks of the DAP3 eCLIP peaks spanning the exon5 to exon6 of *WSB1* gene.

(b) IGV browser tracks of the DAP3 eCLIP peaks spanning the exon5 to exon7 of *SSBP3* gene.

(c) IGV browser tracks of the DAP3 eCLIP peaks spanning the exon1 to exon3 of *RHBDD2* gene.

Significant peaks are marked by blue and green bars.

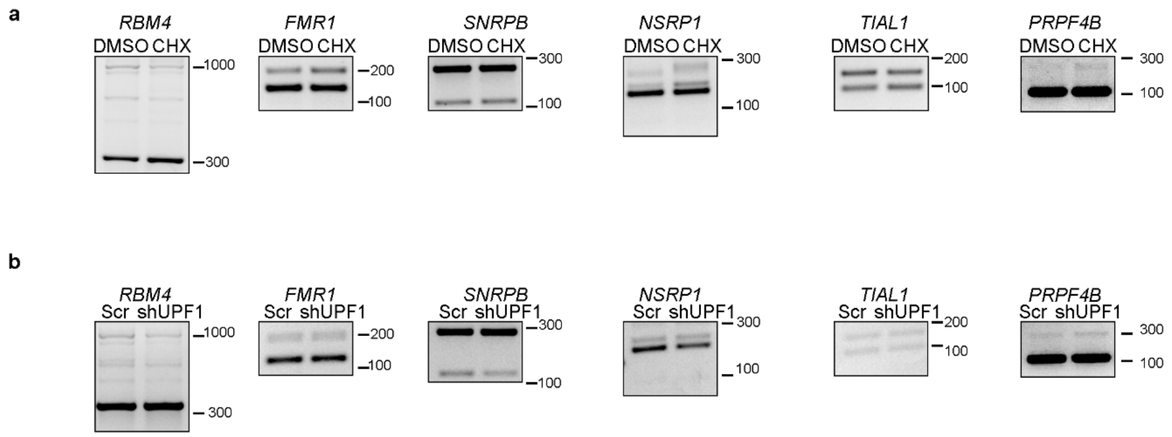

Supplementary Figure 8

Supplementary Figure 8. Changes in splicing pattern of DAP3 target genes *RBM4, FMR1, SNRPB, NSRP1, TIAL1* and *PRPF4B* upon the inhibition of NMD.

(a)Semiquantitative RT-PCR analysis of the *RBM4, FMR1, SNRPB, NSRP1, TIAL1* and *PRPF4B* splicing isoforms upon DMSO or CHX treatment for 6h.

(b)Semiquantitative RT-PCR analysis of the *RBM4, FMR1, SNRPB, NSRP1, TIAL1* and *PRPF4B* splicing isoforms after transfection with the scramble control or shUPF1 for 48h.

(a, b) Representative images of n=2 biologically independent samples. Marker unit: bp.

Source data are provided in Source Data file.

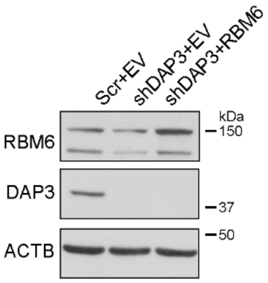

Supplementary Figure 9

Supplementary Figure 9. Rescue of DAP3-mediated RBM6 downregulation by reintroduction of RBM6.

Western blot analysis of RBM6, DAP3 and β-actin (ACTB) protein expression in the indicated stable EC109 cells. Source data are provided in Source Data file.

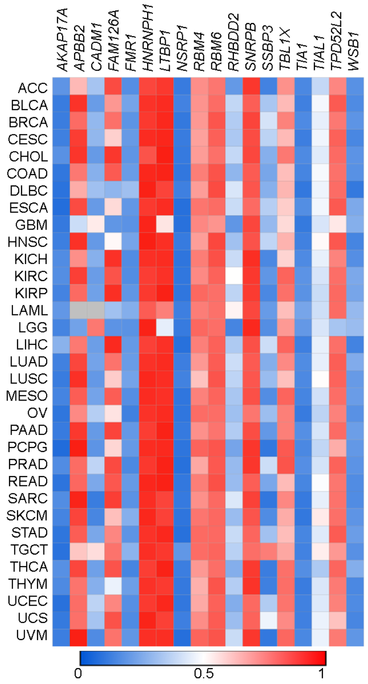

Supplementary Figure 10

Supplementary Figure 10. Analysis of DAP3-modulated splicing events in 33 types of cancers, from the TCGA Spliceseq dataset.

Heatmap showing 18 experimentally validated DAP3-modulated splicing events in 33 TCGA cancer types. The color spectrum indicates average PSI values for each tumor type. Source data are provided in Source Data file.

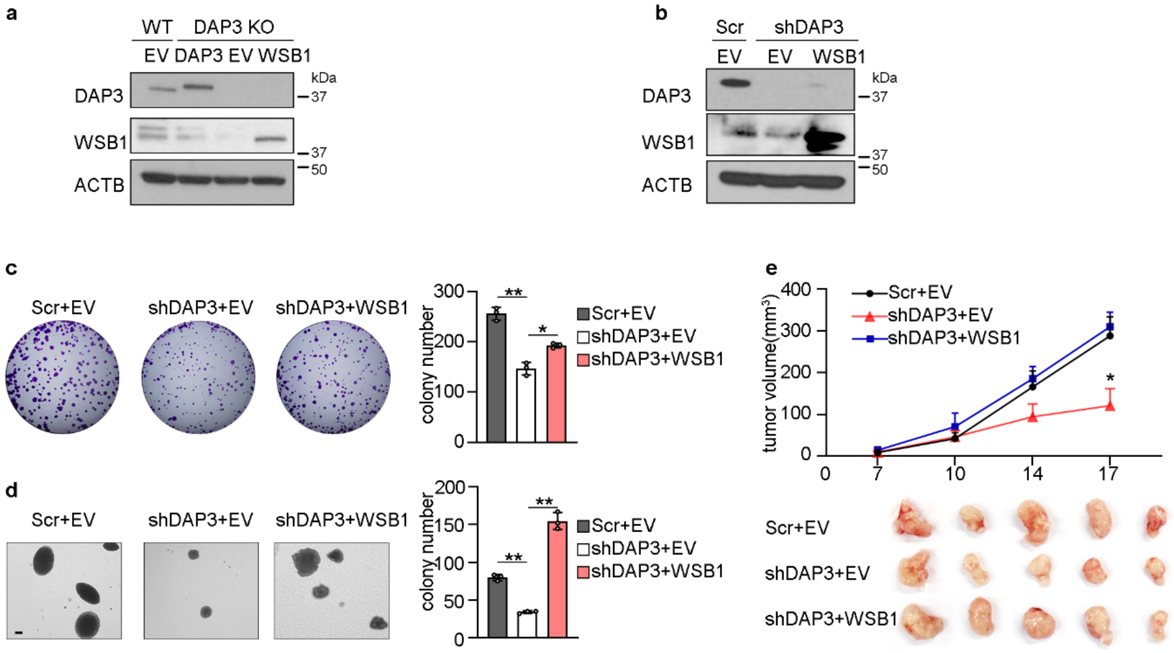

Supplementary Figure 11

Supplementary Figure 11. Restoring WSB1 expression in *DAP3* KO cells rescued the suppressed tumorigenicity.

(a) Western blot analysis of DAP3 and WSB1 protein expression in *DAP3* KO EC109 cells with restored DAP3 expression.

(b) Western blot analysis of DAP3 and WSB1 protein expression in *DAP3* KD EC109 cells that were overexpressed with WSB1 expression construct.

(c,d) Quantification of foci formation (c) or soft agar colony formation (d) induced by the indicated stable cells. Scale bar: 200µm. Data are represented as mean ± s.d. of n=3 biologically independent wells. Statistical significance is determined by unpaired, two-tailed Student's t-test (*, p<0.05, **, p<0.01).

(e) Growth curve of tumors derived from the indicated cells in mice, over a 17-day observation period. Tumors derived from the indicated stable cell lines at end point (n = 5 mice per group). Data are presented as the mean ± s.e.m. Statistical significance is determined by unpaired, two-tailed Student's *t*-test (*, p<0.05).

Exact p-values and source data are provided in Source Data file.
