# Supplementary material for: Multilayered control of splicing regulatory networks by DAP3 leads to widespread alternative splicing changes in cancer
Source: Nat Commun. 2022 Apr 4;13:1793. doi: 10.1038/s41467-022-29400-7 (PMC8980049; doi:10.1038/s41467-022-29400-7)
Supplement: Supplementary file 3 — Description of Additional Supplementary Files [file 41467_2022_29400_MOESM3_ESM.pdf]

Description of Additional Supplementary Files

File Name: Supplementary Data 1
Description: Significant DAP3 binding peaks in eCLIP-seq.

File Name: Supplementary Data 2
Description: Significant alternative splicing events after DAP3 knockdown in EC109 cells.

File Name: Supplementary Data 3
Description: Significant alternative splicing events after DAP3 knockdown in KYSE180 cells.

File Name: Supplementary Data 4
Description: SILAC-MS data of DAP3 pulldown in EC109 cells.

File Name: Supplementary Data 5
Description: Significant alternative splicing events co-modulated after DAP3 and RBM6 knockdown in EC109 cells.

File Name: Supplementary Data 6
Description: Significant alternative splicing events in 13 pairs of TCGA ESCA tumors and matched normal tissues.

File Name: Supplementary Data 7
Description: Primer and probe sequences.
