# Supplementary material for: Multilayered control of splicing regulatory networks by DAP3 leads to widespread alternative splicing changes in cancer
Source: Nat Commun. 2022 Apr 4;13:1793. doi: 10.1038/s41467-022-29400-7 (PMC8980049; doi:10.1038/s41467-022-29400-7)
Supplement: Supplementary file 11 — Reporting Summary [file 41467_2022_29400_MOESM11_ESM.pdf]

![nature portfolio]

Corresponding author(s): Leilei Chen/Jian Han

Last updated by author(s): Feb 10, 2022

Reporting Summary

Nature Portfolio wishes to improve the reproducibility of the work that we publish. This form provides structure for consistency and transparency in reporting. For further information on Nature Portfolio policies, see our Editorial Policies and the Editorial Policy Checklist.

Statistics

For all statistical analyses, confirm that the following items are present in the figure legend, table legend, main text, or Methods section.

n/a	Confirmed	
☐	☒	The exact sample size (*n*) for each experimental group/condition, given as a discrete number and unit of measurement
☐	☒	A statement on whether measurements were taken from distinct samples or whether the same sample was measured repeatedly
☐	☒	The statistical test(s) used AND whether they are one- or two-sided<br>*Only common tests should be described solely by name; describe more complex techniques in the Methods section.*
☒	☐	A description of all covariates tested
☒	☐	A description of any assumptions or corrections, such as tests of normality and adjustment for multiple comparisons
☐	☒	A full description of the statistical parameters including central tendency (e.g. means) or other basic estimates (e.g. regression coefficient) AND variation (e.g. standard deviation) or associated estimates of uncertainty (e.g. confidence intervals)
☐	☒	For null hypothesis testing, the test statistic (e.g. *F*, *t*, *r*) with confidence intervals, effect sizes, degrees of freedom and *P* value noted<br>*Give P values as exact values whenever suitable.*
☒	☐	For Bayesian analysis, information on the choice of priors and Markov chain Monte Carlo settings
☒	☐	For hierarchical and complex designs, identification of the appropriate level for tests and full reporting of outcomes
☒	☐	Estimates of effect sizes (e.g. Cohen's *d*, Pearson's *r*), indicating how they were calculated

Our web collection on statistics for biologists contains articles on many of the points above.

Software and code

Policy information about availability of computer code

Data collection	DAP3 eCLIP was sequenced by paired-end 100bp sequencing performed on the Illumina HiSeq 4000 platform;<br>RNA-seq data was generated by stand-specific sequencing performed on the Illumina HiSeq 4000 or Illumina NovaSeq 6000 platform;<br>DAP3-regulated splicing data in TCGA samples was downloaded from TCGA SpliceSeq (http://bioinformatics.mdanderson.org/TCGASpliceSeq)
Data analysis	eCLIP-seq and rMATS analysis on CSI NGS Portal (https://csibioinfo.nus.edu.sg/csingsportal/login/home.php)<br>Homer v2 (http://homer.ucsd.edu/homer/motif/)<br>Metascape v3.5(http://metascape.org);<br>Morpheus (https://software.broadinstitute.org/morpheus);<br>WebGestalt v2019(http://www.webgestalt.org/)<br>ImageJ v1.8.0<br>Graphpad Prism v9.2.0<br>Microsoft Excel 2019

For manuscripts utilizing custom algorithms or software that are central to the research but not yet described in published literature, software must be made available to editors and reviewers. We strongly encourage code deposition in a community repository (e.g. GitHub). See the Nature Portfolio guidelines for submitting code & software for further information.

Data

Policy information about availability of data

All manuscripts must include a data availability statement. This statement should provide the following information, where applicable:

- Accession codes, unique identifiers, or web links for publicly available datasets
- A description of any restrictions on data availability
- For clinical datasets or third party data, please ensure that the statement adheres to our policy

Accession ID: GSE123020 (RNA-Seq) https://www.ncbi.nlm.nih.gov/geo/query/acc.cgi?acc=GSE123020, GSE172078 (RNA-Seq) https://www.ncbi.nlm.nih.gov/geo/query/acc.cgi?acc=GSE172078 and GSE144318 (eCLIP-Seq) https://www.ncbi.nlm.nih.gov/geo/query/acc.cgi?acc=GSE144318.
Databank URL: http://www.ncbi.nlm.nih.gov/geo/.

Field-specific reporting

Please select the one below that is the best fit for your research. If you are not sure, read the appropriate sections before making your selection.

☒ Life sciences ☐ Behavioural & social sciences ☐ Ecological, evolutionary & environmental sciences

For a reference copy of the document with all sections, see nature.com/documents/nr-reporting-summary-flat.pdf

Life sciences study design

All studies must disclose on these points even when the disclosure is negative.

Sample size	All sample size information were stated in figures and figure legends. The sample sizes were determined based on common practice in the field.
Data exclusions	For patient survival analysis, patients with no survival information or no splicing information were excluded.
Replication	Replicate experiments were successful. The number of replication for each experiments were indicated in figure legends and in "Statistics and Reproducibility" section.
Randomization	Cells were randomly allocated into each group in relevant experiments.
Blinding	The investigators were not blinded as proper controls were already included during experiment design.

Reporting for specific materials, systems and methods

We require information from authors about some types of materials, experimental systems and methods used in many studies. Here, indicate whether each material, system or method listed is relevant to your study. If you are not sure if a list item applies to your research, read the appropriate section before selecting a response.

Materials & experimental systems

n/a	Involved in the study	
☐	☒	Antibodies
☐	☒	Eukaryotic cell lines
☒	☐	Palaeontology and archaeology
☐	☒	Animals and other organisms
☐	☒	Human research participants
☒	☐	Clinical data
☒	☐	Dual use research of concern

Methods

n/a	Involved in the study	
☒	☐	ChIP-seq
☒	☐	Flow cytometry
☒	☐	MRI-based neuroimaging

Antibodies

Antibodies used	anti-DAP3 (Abcam ab2637);<br>anti-SFPQ (Santa Cruz sc-271796);<br>anti-NONO (Santa Cruz sc-166702);<br>anti-WSB1 (Novus NBP2-82049);<br>anti-ATM (Santa Cruz sc-377293);<br>anti-RBM6 (Santa Cruz sc-376201);<br>anti-β-actin (Santa Cruz sc-47778);

anti-mouse IgG, HRP-linked, (Cell Signaling Technology Cat# 7076; RRID:AB_330924);
Anti-rabbit IgG, HRP-linked, (Cell Signaling Technology Cat#7074; RRID:AB_2099233);
IgG (Invitrogen, 02-6202)

Validation

Antibodies were validated by the respective vendors and knockdown or overexpression of respective proteins in this study.
anti-DAP3 (Abcam ab2637): Han, Jian, et al. "Suppression of adenosine-to-inosine (A-to-I) RNA editome by death associated protein 3 (DAP3) promotes cancer progression." Science advances 6.25 (2020): eaba5136.
anti-SFPQ (Santa Cruz sc-271796): Zhang, Pengfei, et al. "The lncRNA Neat1 promotes activation of inflammasomes in macrophages." Nature communications 10.1 (2019): 1-17.
anti-NONO (Santa Cruz sc-166702): Yamamoto, Keita, et al. "A histone modifier, ASXL1, interacts with NONO and is involved in paraspeckle formation in hematopoietic cells." Cell Reports 36.8 (2021): 109576.
anti-WSB1 (Novus NBP2-82049): https://www.novusbio.com/products/wsb1-antibody_nbp2-82049
anti-ATM (Santa Cruz sc-377293): Zhu, Songli, et al. "Kinesin Kif2C in regulation of DNA double strand break dynamics and repair." Elife 9 (2020): e53402.
anti-RBM6 (Santa Cruz sc-376201): Liu, Huina, et al. "LncRNA, PLXDC2-OT promoted the osteogenesis potentials of MSCs by inhibiting the deacetylation function of RBM6/SIRT7 complex and OSX specific isoform." Stem Cells 39.8 (2021): 1049-1066.

Eukaryotic cell lines

Policy information about cell lines

Cell line source(s)

EC109 and KYSE180 were kindly provided by Professor Xin-Yuan Guan (Department of Clinical Oncology, The University of Hong Kong).

Authentication

None of the cell lines used were authenticated.

Mycoplasma contamination

Mycoplasma contamination was not found in these cell lines.

Commonly misidentified lines
(See ICLAC register)

None such line was used.

Animals and other organisms

Policy information about studies involving animals; ARRIVE guidelines recommended for reporting animal research

Laboratory animals

NOD scid gamma (NSG) mice (The Jackson Laboratory, RRID:IMSR_JAX:005557) were maintained in pathogen–free (SPF) facility in NUS Comparative Medicine Department. Less than 5 mice with same sex were housed in a cage at 20-25 °C and 50% humidity with a 12 h light/dark cycle. 4- to 6-week-old NOD scid gamma (NSG) mice (Fig 7j: n=3 males and n=3 females for each group; Supplementary Fig. 11e: n=2 males and n=3 females for each group) were used.

Wild animals

No wild animal was used.

Field-collected samples

No field collection.

Ethics oversight

All animal experiments were approved by and performed in accordance with the Institutional Animal Care and Use Committees of National University of Singapore (R16-1644).

Note that full information on the approval of the study protocol must also be provided in the manuscript.

Human research participants

Policy information about studies involving human research participants

Population characteristics

All human participants data were obtained from The Cancer Genome Atlas (TCGA) Program (https://www.cancer.gov/tcga).

Recruitment

Recruitment of human participants was previously described by TCGA Research Network (https://www.cancer.gov/tcga).

Ethics oversight

Ethics oversight of the TCGA program is provided: https://www.cancer.gov/about-nci/organization/ccg/research/structural-genomics/tcga/history/policies

Note that full information on the approval of the study protocol must also be provided in the manuscript.
